# Alveolar Type II Epithelial Cells Contribute to the Anti-Influenza A Virus Response in the Lung by Integrating Pathogen- and Microenvironment-Derived Signals

**DOI:** 10.1128/mBio.00276-16

**Published:** 2016-05-03

**Authors:** S. Stegemann-Koniszewski, Andreas Jeron, Marcus Gereke, Robert Geffers, Andrea Kröger, Matthias Gunzer, Dunja Bruder

**Affiliations:** a Immune Regulation, Helmholtz Centre for Infection Research, Braunschweig, Germany; b Infection Immunology, Institute of Medical Microbiology, Infection Control and Prevention, Otto-von-Guericke University, Magdeburg, Germany; c Genome Analytics, Helmholtz Centre for Infection Research, Braunschweig, Germany; d Innate Immunity and Infection, Helmholtz Centre for Infection Research, Braunschweig, Germany; e Molecular Microbiology, Institute of Medical Microbiology, Infection Control and Prevention, Otto-von-Guericke University, Magdeburg, Germanye; f Institute of Experimental Immunology and Imaging, University of Duisburg-Essen, Essen, Germany

## Abstract

Influenza A virus (IAV) periodically causes substantial morbidity and mortality in the human population. In the lower lung, the primary targets for IAV replication are type II alveolar epithelial cells (AECII), which are increasingly recognized for their immunological potential. So far, little is known about their reaction to IAV and their contribution to respiratory antiviral immunity *in vivo*. Therefore, we characterized the AECII response during early IAV infection by analyzing transcriptional regulation in cells sorted from the lungs of infected mice. We detected rapid and extensive regulation of gene expression in AECII following *in vivo* IAV infection. The comparison to transcriptional regulation in lung tissue revealed a strong contribution of AECII to the respiratory response. IAV infection triggered the expression of a plethora of antiviral factors and immune mediators in AECII with a high prevalence for interferon-stimulated genes. Functional pathway analyses revealed high activity in pathogen recognition, immune cell recruitment, and antigen presentation. Ultimately, our analyses of transcriptional regulation in AECII and lung tissue as well as interferon I/III levels and cell recruitment indicated AECII to integrate signals provided by direct pathogen recognition and surrounding cells. *Ex vivo* analysis of AECII proved a powerful tool to increase our understanding of their role in respiratory immune responses, and our results clearly show that AECII need to be considered a part of the surveillance and effector system of the lower respiratory tract.

## IMPORTANCE

In order to confront the health hazard posed by IAV, we need to complete our understanding of its pathogenesis. AECII are primary targets for IAV replication in the lung, and while we are beginning to understand their importance for respiratory immunity, the *in vivo* AECII response during IAV infection has not been analyzed. In contrast to studies addressing the response of AECII infected with IAV *ex vivo*, we have performed detailed gene transcriptional profiling of AECII isolated from the lungs of infected mice. Thereby, we have identified an exceptionally rapid and versatile response to IAV infection that is shaped by pathogen-derived as well as microenvironment-derived signals and aims at the induction of antiviral measures and the recruitment and activation of immune cells. In conclusion, our study presents AECII as active players in antiviral defense *in vivo* that need to be considered part of the sentinel and effector immune system of the lung.

## INTRODUCTION

Influenza A virus (IAV) still poses a serious threat to human health, and a detailed understanding of IAV pathogenesis is essential to adequately confront this hazard. IAV infections are primarily restricted to the respiratory tract, where epithelial cells, alveolar macrophages (AM), and dendritic cells (DC) trigger the first innate responses (1). IAV bears ligands for several pathogen recognition receptors (PRR), and the main triggered PRR are Toll-like receptor 3 (TLR3) and TLR7 as well as RIG-I, MDA5, and the NLRP3 inflammasome (1). These are engaged in the antiviral response in a cell-type-specific manner (2, 3). Via partly redundant signaling pathways, PRR ligation leads to the activation of effector mechanisms comprised of type I/III interferons (IFNs), inflammatory mediators, antimicrobial effectors, and signals inducing adaptive immunity. In general, viral infections are marked by the strong release of type I interferons. These trigger the expression of a multitude of interferon-stimulated genes (ISG) through the ubiquitously expressed IFN-α/β receptor (IFNAR) (2). ISG expression is also induced through IFN-λ (IFN III), which is released during IAV infection and is sensed through the interleukin-28 (IL-28) receptor α (IL-28Rα) primarily expressed by epithelial cells of the respiratory tract and gut (4).

In the lower respiratory tract, the lining epithelium is comprised of alveolar type I and type II epithelial cells (AECI and AECII, respectively), and at this site, AECII are the main target cells for IAV replication (5, 6). AECII cover about 5% of the alveolar surface, while they comprise about 60% of the alveolar lining cells and 15% of the parenchymal cells (7). Until recently, the secretion of surfactant, the maintenance of the mechanical barrier, and the provision of constitutive antimicrobial defense were conceived as their main functions (7, 8). Beyond these, we are only beginning to understand the potential of AECII to regulate respiratory immune responses in autoimmunity and infection (9–12).

Since AECII are primary targets for viral replication in the lower lung and actively contribute to pulmonary immunity, it is likely that they influence efficient host responses directed at IAV. A number of studies have addressed the response of AECII to IAV *in vitro* and showed them to express functional PRR and to produce cytokines and chemokines (13–17). The nature and the relevance of the AECII response to IAV, however, lack ultimate clarification, as these studies were performed using cell lines or primary cells infected in culture. Little is known about the AECII response to IAV infection *in vivo* and how this contributes to the respiratory immune reaction. To overcome these limitations, we characterized the *in vivo* response of AECII to IAV infection by analyzing primary AECII from the lungs of infected mice*.*

## RESULTS

### IAV infection triggers AECII-specific transcriptional regulation *in vivo.*

**FIG 1:**
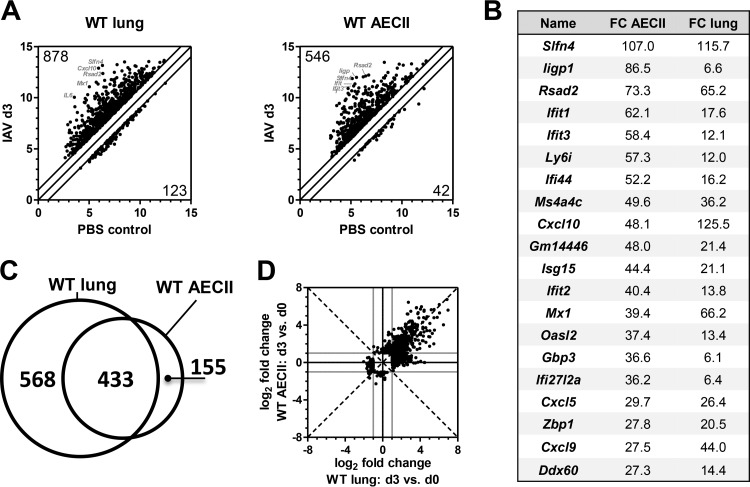
Respiratory IAV infection triggers AECII-specific transcriptional regulation. Mice were infected with IAV or treated with PBS and sacrificed 3 days later. Total RNA from whole lungs (*n* = 3 mice for each condition) and sorted AECII (*n* = 2 individual samples for each condition; 5 mice per sample) was subjected to microarray analysis. Data were analyzed by comparing IAV-infected with uninfected control samples. (A) Scatter plots of regulated transcripts with a fold change of ≥±2 (threshold represented by the diagonal lines). Data represent normalized log_2_ signal intensities (averaged over replicates). The number of up-/downregulated transcripts and the gene symbols of the top 5 upregulated transcripts are indicated. (B) List of the 20 most intensely upregulated transcripts in AECII with fold change values for AECII and lung. (C) Comparison of the transcripts identified in panel A with respect to regulation in lung and/or AECII. (D) Scatter plot showing absolute log_2_ fold changes over the respective PBS controls of the transcripts differentially expressed in AECII and/or lung tissue. The dashed bisecting lines indicate equal fold changes. Gray lines indicate the fold change threshold of ±2.

We have previously optimized our IAV infection model for the isolation of pure and viable AECII that express increasing amounts of viral protein over the first days of infection (9, 18). In this model of lethal IAV infection, mice rapidly lose weight, the virus replicates efficiently in the lungs, and high copy numbers of the viral genome are detected in the isolated AECII (see Fig. S1 in the supplemental material). Typically, inflammatory mediators and immune cell recruitment to the respiratory tract are induced by day 3 postinfection (19), and we analyzed transcriptional regulation in AECII and lung tissue at this time point. For AECII, two independent microarray experiments were conducted for each condition and the material for each independent experiment was pooled from five infected animals. For lung tissue, three independent microarrays were performed for each condition and each array represents an independent animal. Fold change (FC) values of 2 or more over controls were considered indicative of up- or downregulation. In lung tissue, 878 transcripts were upregulated and 123 were downregulated following IAV infection. Extensive transcriptional regulation was also detected in AECII, with 546 upregulated and 42 downregulated transcripts (Fig. 1A). In the lung, the 5 most intensely upregulated transcripts were interleukin-6 (IL-6) (FC = 90), CXCL10, and the IFN-induced proteins Mx1 and Rsad2 as well as Slfn4, which has been suggested to be IFN I inducible (20). There was an overlap with AECII, where Rsad2 and Slfn4, along with the IFN-induced proteins Ifit1, Ifit3, and Iigp1, were among the top 5 most regulated transcripts, indicating a prominent role for IFNs in the AECII response (Fig. 1B). The intense upregulation of CXCL5, CXCL9, and CXCL10 (Fig. 1B) in AECII as well as the large overlap of the transcripts found regulated in either lungs or AECII (Fig. 1C) pointed at a strong potential of AECII to contribute to respiratory immunity. In order to confirm the AECII transcriptional regulation following *in vivo* IAV infection detected by the microarray analyses, the expression of a selection of these transcripts was analyzed by quantitative real-time PCR. Indeed, this approach confirmed the significant upregulation of *CXCL5*, *CXCL10*, *IFIT2*, *IRF7*, *MX2*, and *USP18* (see Fig. S2). Overall, the detection of AECII-specific transcripts showed that AECII most likely serve cell-type-specific functions. These transcripts were associated with Gene Ontology (GO) terms such as defense response (Bonferroni corrected *P* value, 2.8 × 10^−12^), innate immune response (corrected *P* value, 0.00013), and cytokine production (corrected *P* value, 0.00077). The quality, i.e., whether up- or downregulation occurred, of transcriptional regulation in AECII and lung tissue was not changed between the two sample sets (Fig. 1D). Overall, these results clearly demonstrated that AECII strongly react to IAV infection *in vivo* and hold strong potential to contribute to the respiratory immune response. To characterize this contribution in greater detail, we performed AECII transcriptional analyses over the early course of infection.

### Early transcriptional regulation in AECII follows distinct kinetics.

**FIG 2:**
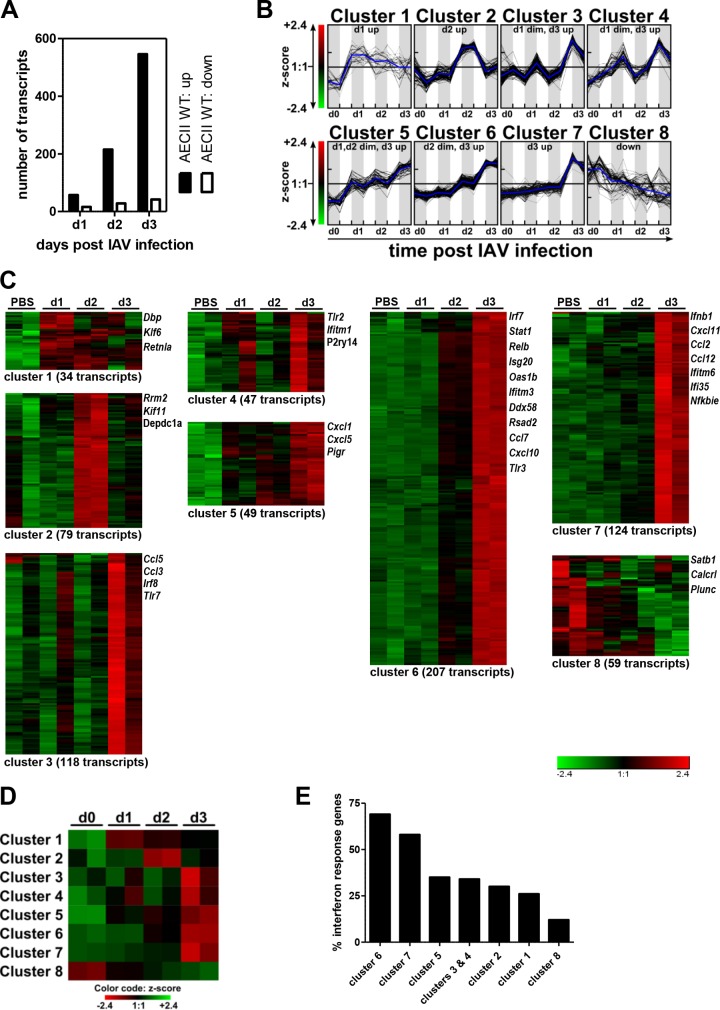
Kinetics of transcriptional regulation in WT AECII in the early course of IAV infection. Mice were infected with IAV and sacrificed 1, 2, or 3 days later. RNA from sorted AECII (*n* = 2 individual samples for each condition; 5 mice per sample) was subjected to microarray analysis. For each time point, microarray data were compared to the uninfected control. (A) The number of transcripts up-/downregulated with a fold change of ≥±2 in AECII. (B) k-means cluster analysis of the transcripts differentially regulated with a fold change of ≥±2 in AECII on day 1, 2, or 3. Data were transformed into Z scores. Line plots show the Z scores of the transcripts of the individual k-means clusters over time (for both replicates per time point). The blue lines indicate the average Z score. (C) Heat map visualization of the Z scores in the individual k-means clusters. The number of transcripts and selected members are indicated for each cluster. (D) Heat map visualization of the average Z score value per column of the k-means clusters. (E) Percent interferon response genes within the individual k-means clusters as determined using the Interferome v2.01 database.

Over the first 3 days postinfection, the number of transcripts differentially regulated in AECII strongly increased and the number of downregulated transcripts was considerably lower than the number of those upregulated. This suggested that AECII were increasingly stimulated over time and that activation rather than suppression of gene transcription dictated their response (Fig. 2A). k-means clustering was performed (Fig. 2B to D), and the resulting clusters included transcripts upregulated exclusively only on day 1, 2, or 3 and transcripts downregulated over time. Most differentially expressed transcripts were upregulated on day 3 postinfection, and these further segregated into transcripts (i) slightly upregulated on day 1, (ii) slightly upregulated on days 1 and 2, and (iii) slightly upregulated from day 2 onward (Fig. 2C and D). As IAV infection induces the rapid release of IFN I/III, we assessed the prevalence of ISG within the clusters using the Interferome v2.01 database (21). By far, the highest proportion of ISG was present in clusters 6 and 7, pointing at a strong and progressive AECII response to IFN I and/or IFN III (Fig. 2E).

### TLR7 deficiency alters the regulation of gene expression in AECII.

**FIG 3:**
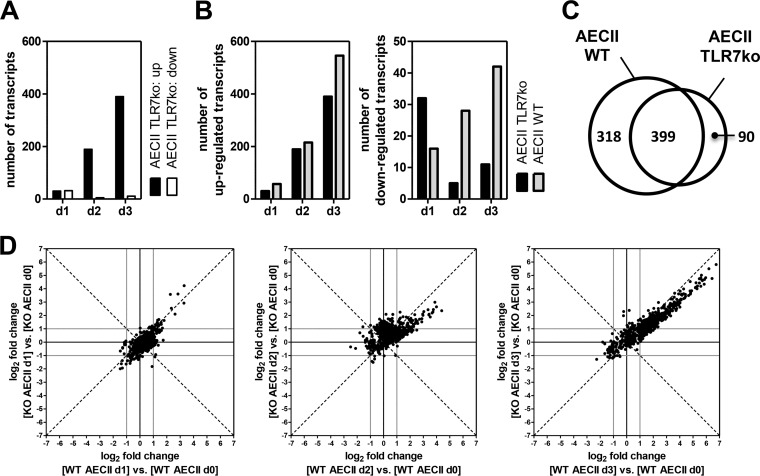
Transcriptional regulation in response to IAV infection is blunted in AECII isolated from TLR7-deficient hosts. Wild-type (WT) and TLR7ko mice were infected with IAV and sacrificed 1, 2, or 3 days postinfection. RNA from sorted AECII (*n* = 2 individual samples per time point; 5 mice per sample) was subjected to microarray analysis. For each time point, microarray data were compared to the respective uninfected control. (A) Number of transcripts up- or downregulated with a fold change of ≥±2 in TLR7ko AECII in the course of IAV infection. (B) Comparison between the number of up- and downregulated transcripts in WT and TLR7ko AECII. (C) Venn diagram comparing the transcripts regulated in AECII on day 1, 2, and/or 3 post-IAV infection with regard to their regulation in WT AECII, TLR7ko AECII, or both. (D) Scatter plots displaying the absolute log_2_ fold changes of the transcripts regulated more than 2-fold (up or down) in WT and/or TLR7ko AECII on days 1, 2, and/or 3 post-IAV infection. The dashed bisecting lines indicate equal fold changes. Gray lines indicate the fold change threshold of ±2.

We hypothesized that the host relies on IAV-sensing PRR in order to mount a full AECII response and analyzed transcriptional regulation in lungs and AECII of TLR7-knockout (TLR7ko) mice. On day 3 post-IAV infection, considerable transcriptional regulation was detected (see Fig. S3 in the supplemental material). Over time, AECII were increasingly activated also in TLR7ko mice (Fig. 3A). However, the number of upregulated transcripts was clearly reduced in comparison to wild-type (WT) AECII (Fig. 3B), whereas the comparison of gene transcription levels in AECII isolated from uninfected WT and TLR7ko mice did not yield differences in baseline expression (see Fig. S3 in the supplemental material). As there was a large overlap with the transcripts regulated in the WT, TLR7ko AECII did not harbor an independent gene expression profile (Fig. 3C). The 90 transcripts exclusively regulated in TLR7ko AECII were not significantly associated with any annotated Gene Ontology (GO) terms, whereas the transcripts regulated only in WT AECII showed significant association with GO terms such as innate immune response (Bonferroni corrected *P* value, 2.68 × 10^−21^), immune effector process (1.38 × 10^−18^), and defense response to virus (2.28 × 10^−15^). A list of the most intensely regulated of these TLR7-dependent transcripts is provided in Table S1 in the supplemental material. In addition, the fold changes of the vast majority of the transcripts upregulated in both mouse strains were lower in TLR7ko AECII throughout the early course of infection (Fig. 3D). Therefore, the AECII response of TLR7-deficient hosts during IAV infection was blunted regarding both the number of differentially expressed transcripts and the degree of fold change regulation, demonstrating the importance of a single PRR for the full AECII response.

### IAV infection triggers transcriptional regulation of a multitude of immunological factors.

We performed pathway analyses on the transcripts differentially expressed in AECII on day 3 post-IAV infection using Ingenuity pathway analysis (IPA). The most significantly overrepresented pathways clearly indicated strong immunological activity, as they were involved in pathogen recognition, the induction and shaping of immune responses, and immune cell recruitment (Table 1). Increasing *P* values over time illustrated increasing relevance in the AECII response. These results clearly demonstrated that AECII exert pronounced immunological functions in IAV infection *in vivo*. Of note, the functional pathways overrepresented in the transcripts regulated in TLR7ko AECII were largely identical to those identified for WT AECII (see Table S2 in the supplemental material).

**TABLE 1:** Functional pathways most significantly overrepresented in the transcripts differentially expressed in AECII^a^

Ranking by *P* value	Canonical pathway	AECII day 1	AECII day 2	AECII day 3
1	Communication between innate and adaptive immune cells	1.53	2.05	20.49
2	Role of pattern recognition receptors in recognition of bacteria and viruses		3.03	19.04
3	Granulocyte adhesion and diapedesis	2.53	3.31	15.44
4	Cross talk between dendritic cells and natural killer cells			14.79
5	Dendritic cell maturation			14.33
6	TREM1 signaling	1.73	2.42	13.79
7	Agranulocyte adhesion and diapedesis	2.44	3.14	12.91
8	Altered T cell and B cell signaling in rheumatoid arthritis			12.79
9	Interferon signaling		4.56	12.64
10	Antigen presentation pathway		2.34	11.66

a The 10 pathways most significantly overrepresented in the transcripts differentially regulated in WT AECII on day 3 postinfection are listed. Pathways were ranked by Fisher exact test *P* value. The data columns indicate the −log(*P* value) for overrepresentation of the respective pathways for all microarray data sets (WT AECII; days 1, 2, and 3 postinfection), listing only those values indicating statistical significance (*P* < 0.05).

**FIG 4:**
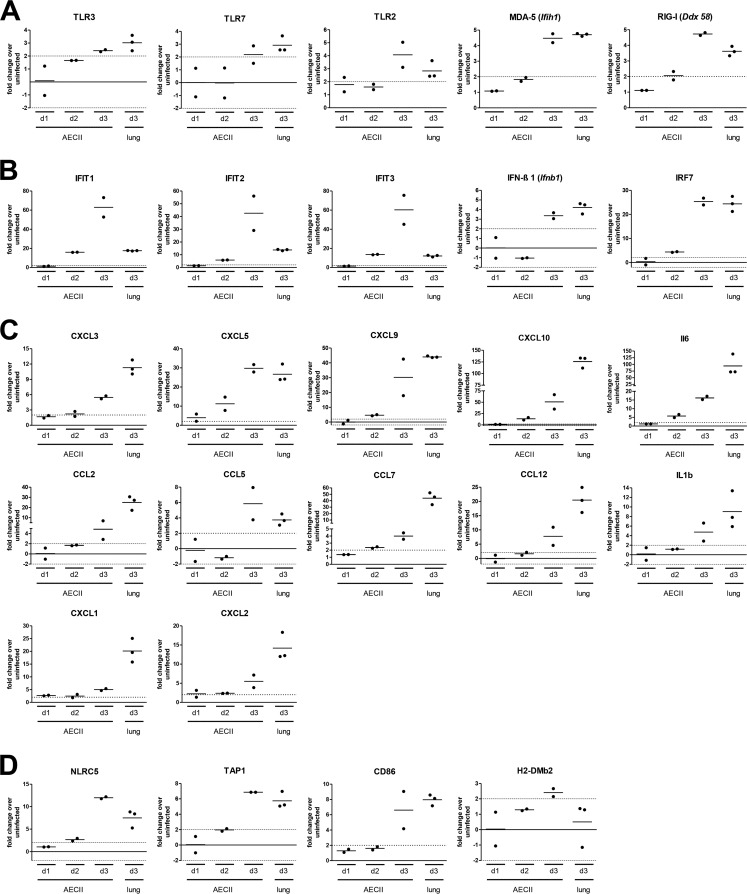
IAV infection triggers the differential expression of a multitude of molecules involved in antimicrobial defense. The graphs depict the fold change regulation of selected transcripts as determined by microarray analysis of WT AECII and lungs isolated at the indicated time points post-IAV infection. Data are shown as the mean and the individual results from two independent replicate microarray experiments (2 independent samples; 5 mice per sample) for AECII and three independent replicate microarray experiments for lung tissue (three independent samples). The transcripts listed are grouped into those encoding pathogen recognition receptors (A), factors associated with the IFN I/III response (B), cytokines and chemokines (C), and factors associated with antigen presentation (D). For each bar graph, the dashed horizontal line indicates a fold change of 2.

We furthermore evaluated transcriptional regulation of key antiviral and immunological factors within the transcript differentially regulated in AECII. Transcription of several, mainly nucleic acid-sensing PRR was upregulated in AECII in the course of IAV infection (Fig. 4A). Fold change regulation in AECII peaked on day 3 and was similar to or even higher than that detected for whole-lung tissue.

IFIT (IFN-induced protein with tetratricopeptide repeats) 1 to 3, all of which contribute to antiviral defense (22), showed exceptionally strong induction in AECII (Fig. 4B) that by far exceeded that in lung tissue. Interestingly, out of the genes coding for IFN I and IFN III only *Ifnb1* was differentially upregulated in AECII (Fig. 4B). In contrast, also the transcription of interferon regulatory factor 7 (IRF7), which induces IFN and ISG expression (1), was extensively regulated in WT AECII (Fig. 4B).

Also, the transcription of chemokines and cytokines was efficiently induced in AECII, underlining their immunological potential in respiratory infection (Fig. 4C). The extent of upregulation increased over time, and for most cytokines, upregulation was more pronounced in lungs than in AECII. Of note, however, transcription of *Cxcl5*, *Cxcl9*, and *Cxcl10* was upregulated more than 25-fold in AECII and *Cxcl5* and *Ccl5* upregulation in AECII exceeded that in lung tissue.

Next to the secretion of immunological mediators, AECII are capable of presenting antigen on major histocompatibility complex class I (MHC-I) and MHC-II molecules as well as providing costimulation (10, 23–25). Indeed, antigen presentation was among the most significantly enriched pathways in the transcripts differentially expressed in AECII, and the transcriptional regulator of the MHC-I complex NLRC5 (NOD-like receptor family CARD domain-containing 5) as well as Tap1 (transporter associated with antigen processing 1) was upregulated in AECII to a larger extent than in lung tissue (Fig. 4D). Additionally, we detected the upregulation of CD86 as well as H2-DMb2 in AECII following IAV infection.

For the selected factors, transcriptional regulation in TLR7ko AECII showed similar kinetics but in the majority of cases did not reach the magnitude of upregulation observed in WT AECII (see Fig. S4 in the supplemental material). Taken together, AECII reacted to respiratory IAV infection by the differential expression of a multitude of molecules involved in the induction and shaping of immune responses, demonstrating their exceptionally high potential to contribute to respiratory immunity *in vivo*.

### AECII transcriptional regulation correlates with PMN recruitment and IFN I/III levels.

**FIG 5:**
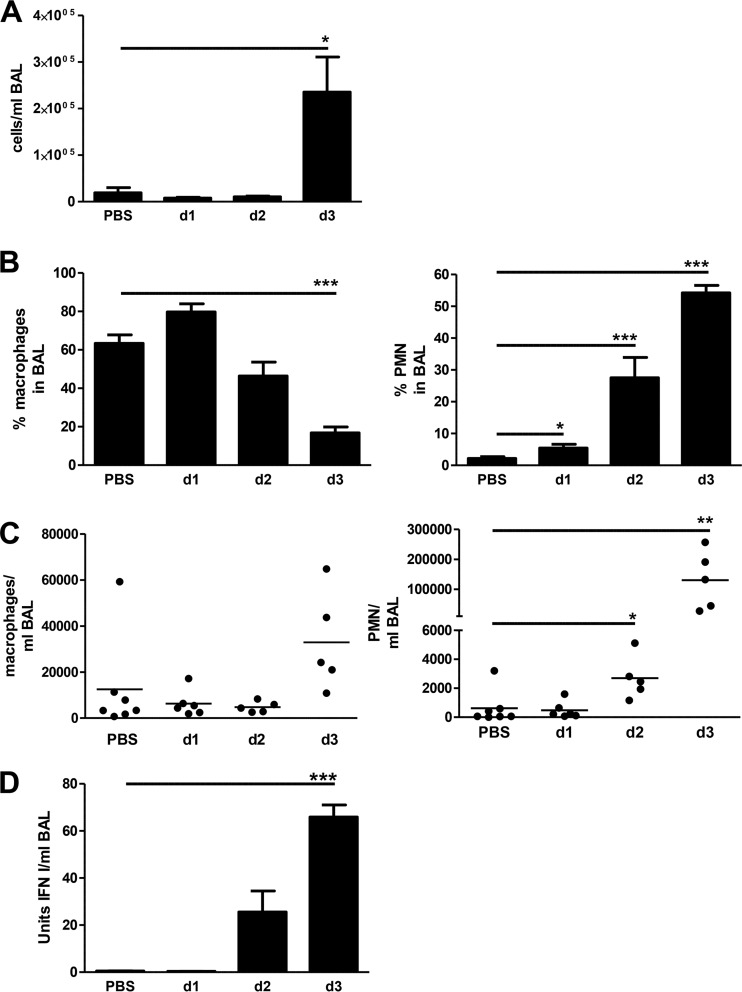
The kinetics of cell recruitment and type I/III interferon levels correlate with AECII transcriptional profiles. WT mice were sacrificed at the indicated time points post-IAV infection. Bronchoalveolar lavage (BAL) fluid cells were counted (A), and the macrophage and polymorphonuclear cell (PMN) populations (B) were assessed by flow cytometry. Cell populations were analyzed by gating on macrophages (F4/80^+^ cells) within all acquired cells and gating on PMN (Gr-1^+^/CD11b^+^) within the F4/80^−^ cell fraction. (C) Cell numbers were calculated from the absolute cell count and percent population for all analyzed individual mice. Data from individual mice and the mean per group are shown. (D) The concentration of bioactive type I IFN in BAL fluid was assessed using IFN I/III-sensitive reporter cells and an IFN-β standard. Data are shown as means ± standard errors of the means or as individual mice and mean per group. All data are compiled from *n* ≥ 5 mice out of at least two independent experiments. Groups were compared by unpaired, two-sided *t* test (*P* values: *, <0.05; **, <0.005; ***, <0.001).

AECII most likely take part in the recruitment of immune effector cells to the respiratory tract. We determined the overall cellularity of bronchoalveolar lavage (BAL) fluid, and a strong and significant increase in cell numbers was detected on day 3 postinfection (Fig. 5A). Since the majority of the chemokines differentially expressed in AECII act as chemoattractants for macrophages and other myeloid cells, mainly polymorphonuclear neutrophils (PMN), we determined the macrophage and PMN populations. In uninfected mice, macrophages were the main cell type present and their relative contribution was significantly reduced by day 3 (Fig. 5B). At the same time, there was a significant increase in the PMN population size and the absolute PMN number (Fig. 5B and C). The kinetics of PMN recruitment and AECII transcriptional regulation of chemokines suggest that AECII contribute to the attraction of innate effectors to the lung. Of note, the blunted transcriptional regulation of chemokines in TLR7-deficient hosts was in line with a delayed recruitment of PMN (see Fig. S5 in the supplemental material).

In order to link ISG induction to interferon levels in the lung, we assessed the levels of bioactive IFN I/III in BAL fluid. A substantial increase in IFN I/III activity was detected on day 2 post-IAV infection, with a significant increase by day 3 (Fig. 5C) which directly correlated with the massive induction of ISG in AECII by day 3 postinfection. Of note, IFN I/III levels were significantly reduced in TLR7-deficient mice (see Fig. S5 in the supplemental material). Taken together, these findings imply a critical role for IFN I/III for the AECII response following IAV infection.

## DISCUSSION

Nearly all of the research that has addressed the response of AECII to IAV was performed in cells infected with the virus *ex vivo* (13–17), whereas *in vivo* studies mostly did not differentiate between different cell types of the epithelium (26).

Our analyses revealed that AECII strongly react to respiratory IAV infection *in vivo*. The response was exceptionally versatile and included differential expression of a plethora of factors associated with antiviral activity and the induction of immune responses. Several antiviral proteins were among the most intensely upregulated transcripts, and the importance of their epithelial expression was highlighted by the finding that fold change induction in AECII often exceeded that in lung tissue. Deficiency in IFITM3, which was upregulated 12-fold in AECII and only 2-fold in lung tissue on day 3 (data not shown), leads to enhanced viral titers and increased mortality in mice, and patients with *IFITM3* mutations exhibit compromised IAV restriction (27). Also, ISG15 and Gbp3 (guanylate-binding protein 3) contribute specifically to the anti-IAV host defense (28, 29). Out of the genes encoding IFN I/III, only *Ifnb1* was upregulated in AECII following *in vivo* IAV infection. This was surprising at first, especially as AECII are described to produce IFN I/III in response to IAV both *in vivo* and *in vitro* (13, 30). However, it has been shown that during *in vivo* IAV infection IFN-β is predominantly produced not by the epithelium but by CD11c-expressing cell populations (31). Furthermore, the depletion of plasmacytoid dendritic cells (pDC) was reported to lead to a significantly diminished IFN I production in the lungs of IAV-infected mice (32). Therefore, we believe that also in our model resident and newly recruited pDC are most likely the main producers of IFN I. This is well in line with the significantly decreased IFN I/III production in TLR7ko mice, as pDC are known to depend on TLR7 (33).

Extensive transcriptional regulation of *CXCL5*, *CXCL9*, and *CXCL10* demonstrated how broadly AECII act on pulmonary host defense. Even though upregulation of most cytokines in lung tissue exceeded that in AECII, upregulation of *CXCL5* and *CCL5* in AECII was stronger than in lung tissue. Blocking of CCL5 in respiratory viral infection reduces the recruitment of CD4^+^ and CD8^+^ T cells (34), and AECII are the predominant source of CXCL5 in the lungs of mice treated with lipopolysaccharide (35). Regarding the role of AECII in the recruitment of effector cells, the significantly attenuated PMN recruitment in TLR7ko mice was well in line with the alleviated induction of CXCL5 expression in their AECII. Of note, however, this reduction in PMN recruitment is not necessarily a consequence of the reduced production of chemotactic cytokines only by AECII, as their upregulation was also strongly reduced in whole-lung tissue of TLR7ko mice. Nevertheless, the extensive induction of cytokine and chemokine transcription in AECII pointed at a strong contribution of AECII to the overall lung response to infection *in vivo*.

Next to MHC-I antigen presentation in AECII, there was also evidence for MHC-II presentation and costimulation. In fact, lethal IAV infection induces MHC-II expression on lung epithelial cells (23), and AECII have been observed to present antigen to CD4^+^ T cells in the context of mycobacterial infection (25). Furthermore, we have previously shown that AECII efficiently present antigen to and activate CD4^+^ T cells and that they are able to promote the induction of regulatory T cells (10). Therefore, MHC-I and also MHC-II antigen presentation displays strategies for AECII to shape the lower respiratory tract immune response during IAV infection *in vivo*.

A clear and distinct role for TLR7 in survival following IAV infection has so far been demonstrated only in mice expressing functional Mx1, unlike the commonly used laboratory mouse strains (36). We and others have rather found TLR7 to be involved in the fine-tuning of innate and adaptive anti-IAV responses (19, 37, 38), and in our model of respiratory IAV infection, there was no difference in morbidity and mortality between WT and TLR7-deficient hosts (see Fig. S6 in the supplemental material). Nevertheless, we found the AECII response to IAV to be substantially blunted in the absence of TLR7. As both unchanged and clearly diminished IFN I production have been described in TLR7ko mice (19, 39), it was surprising that TLR7 played such a central role in ensuring a robust early IFN response in our model. Most likely, the reduced levels of IFN I/III in the respiratory tract of TLR7ko mice to a large extent accounted for the blunted AECII response in these mice, especially since many of the regulated transcripts were ISG. Of note, the numbers of AECII isolated from WT and TLR7ko animals were similar (see Fig. S6 in the supplemental material). However, TLR7ko mice displayed a reduction in viral load in lung tissue and the isolated AECII (see Fig. S6 in the supplemental material). IAV has previously been described to depend on TLR7 for efficient replication (39), and this reduction in viral load, even though not significant by day 3, was a likely reason for the blunted IFN I/III and in turn the blunted AECII response in TLR7ko mice. We are not able to dissect the interdependence of viral replication, IFN I/III production, and transcriptional regulation in AECII from our data. Silencing of TLR7 and other IAV sensors such as RIG-I specifically in AECII will be essential to shed light on this issue. Nevertheless, the high number of ISG triggered and the correlation between the AECII response and IFN I/III levels in the lung clearly showed that AECII react to their specific microenvironment *in vivo*. In addition to our findings, cell culture studies have shown that pretreatment of AECII with tumor necrosis factor alpha (TNF-α) and IFN-α greatly enhanced their cytokine and chemokine response to IAV (16). Also, IL-17A and TNF-α synergistically act on CXCL5 expression by AECII *in vitro* and *in vivo* (40).

**FIG 6:**
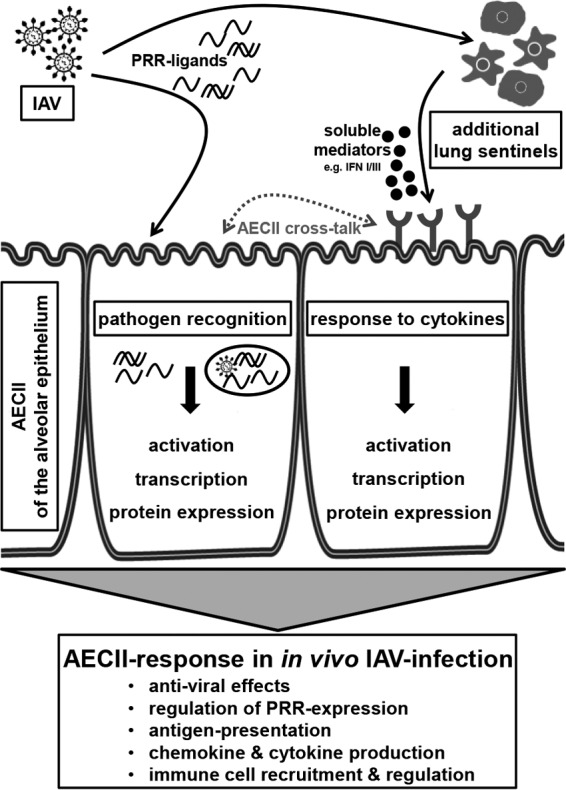
Integrative model of the contribution of AECII to lower respiratory tract anti-IAV responses. The analysis of transcriptional regulation in AECII isolated from infected mice revealed their rapid and versatile immunological response to IAV infection *in vivo*. Based on our results, we propose that AECII integrate signals from a direct interaction with the pathogen with signals provided through cytokines released by additional sentinel cells in order to mount this response.

Ultimately, the detailed contributions and interplay of pathogen recognition and the signals provided by the microenvironment remain a central question for our understanding of AECII activation *in vivo*. Dynamic PRR expression patterns in response to IAV have been observed for AECII (41), and moreover, they respond directly to the virus through various TLR (30, 42). In our study, the exceptionally rapid induction of gene expressional changes and the transcriptional upregulation of nucleic acid-sensing PRR in the course of the infection suggested a direct sensing of the virus by AECII also *in vivo*. Many of the transcripts differentially expressed in AECII are typically triggered by PRR ligation and activation of the NF-κB pathway, and the functional pathway Role of Pattern Recognition Receptors in Recognition of Bacteria and Viruses was the second most significantly overrepresented pathway. Therefore, we propose a model in which the rapid and versatile *in vivo* AECII response following IAV infection is triggered by PRR ligation as well as soluble mediators (Fig. 6). Our results show that the *in vivo* AECII response aims at the inhibition of viral replication and the recruitment and activation of effector cells. Furthermore, we highlight the need to study AECII within their microenvironment to fully characterize their response to pathogens and their role in the local immune system.

## MATERIALS AND METHODS

### Mice.

C57BL/6 mice were purchased from Harlan, and TLR7ko mice (43) (provided by S. Bauer) were bred at the Helmholtz Centre for Infection Research. Eight- to 12-week-old mice were used. Control groups were age and sex matched. All animal experimental procedures were approved by the local government agency (Nds. Landesamt für Verbraucherschutz und Lebensmittelsicherheit, file no. 33.9-42502-04-11/0443).

### Influenza A virus infection.

Madin-Darby canine kidney (MDCK) cell-derived IAV PR8/A/34(H1N1) was obtained as described previously (19). Following intraperitoneal injection of ketamine-xylazine, mice were intranasally infected with 10^1.8^ 50% tissue culture infective doses (TCID_50_) in 40 µl phosphate-buffered saline (PBS).

### AECII isolation.

AECII isolation was performed as described previously (9, 18). Briefly, lungs were perfused and instilled with dispase (5,000 caseinolytic units/100 ml; BD Biosciences) followed by 1% low-melting-point agarose. Excised lungs were incubated in dispase for 45 min. The tissue was disintegrated and incubated for 10 min in Dulbecco's Modified Eagle Medium (DMEM) (Gibco, Life Technologies) supplemented with 650 Kunitz units of bovine DNase I (Sigma-Aldrich) and anti-CD16/32 (2.4G2). Cell suspensions were filtered, and crude cell suspensions from separate mice were pooled. Cells were stained using antibodies for CD16/32 (clone 93), F4/80 (BM8), CD11b (M1/70), CD11c (N418), CD19 (1D3), and CD45 (30-F11). AECII were isolated by sorting for granular sideward scatter^high^ cells negative for the antibody staining using a BD FACSAria instrument.

### Expression microarray analysis.

RNA was isolated from 1 × 10^6^ AECII/sample. For lung tissue, lungs were perfused with PBS, excised, homogenized in RLT buffer (Qiagen), and centrifuged. RNA was isolated from the supernatant using the RNeasy minikit (Qiagen). DNA was digested using the RNase-free DNase set (Qiagen). RNA integrity was tested using the Agilent 2100 Bioanalyzer (Agilent Technologies) with the RNA 6000 Nano/Pico kit (Agilent Technologies). Synthesis and fragmentation of labeled cRNA were performed using the GeneChip 3′ IVT Express kit (Affymetrix). For AECII, two independent microarray experiments were conducted for each condition and the material for each independent experiment was pooled from five infected animals. For lung tissue, three independent microarrays were performed for each condition and each array represents an independent animal. All samples were hybridized to GeneChip Mouse Genome 430 2.0 microarrays (Affymetrix) and stained according to the manufacturer’s recommendations. Microarrays were scanned with an Affymetrix GCS 3000 scanner running with GCOS v1.1.1 software. Every analyzed gene is represented by 16 independent probe pairs which establish the basis for the statistical evaluation. Therefore, only reproducibly regulated genes are included in the analysis.

### Data analysis.

Microarray data were analyzed using GeneSpring GX (Agilent Technologies). The data were summarized, log_2_ transformed, and normalized with the Robust Multi-array Analysis (RMA) algorithm. To exclude probe sets with consistently low signal intensities in all microarrays performed, only probe sets with signal intensities above the 20th percentile in at least one of all the performed microarrays were retained. The signal intensities of the replicate arrays from lungs and AECII were averaged, and fold changes were calculated in reference to respective uninfected lung/AECII controls. With respect to the analyzed sample material, in total 8 comparative conditions were considered for fold change calculation, i.e., [IAV-WT-lung-d3] versus [PBS-WT-lung-d0], [IAV-TLR7ko-lung-d3] versus [PBS-TLR7ko-lung-d0], [IAV-WT-AECII-d1] versus [PBS-WT-AECII-d0], [IAV-WT-AECII-d2] versus [PBS-WT-AECII-d0], [IAV-WT-AECII-d3] versus [PBS-WT-AECII-d0], [IAV-TLR7ko-AECII-d1] versus [PBS-TLR7ko-AECII-d0], [IAV-TLR7ko-AECII-d2] versus [PBS-TLR7ko-AECII-d0], and [IAV-TLR7ko-AECII-d3] versus [PBS-TLR7ko-AECII-d0]. Depending on the type of the biological question, only certain combinations of the 8 comparative conditions were considered, i.e., comparison of lungs versus AECII in WT mice; comparison of lungs versus AECII in TLR7ko mice; comparison of WT AECII d1, d2, and d3 versus d0; and comparison of TLR7ko AECII d1, d2, and d3 versus d0. In each analysis, a fold change of >±2 in at least one out of the regarded conditions was considered indicative for a gene to be up- or downregulated. Fold change calculation considering all 8 comparative conditions results in 1,815 regulated probe sets. Signal intensities of regulated genes were further analyzed by k-means clustering, using the individual microarray replicates and using the Genesis software 1.7.3 (44) following Z score transformation (45). Gene Ontology and pathway analysis was performed with Qiagen’s Ingenuity pathway analysis tool (Ingenuity Systems).

### Detection of IFN I/III in bronchoalveolar lavage (BAL) fluid.

Lungs were flushed with 1 ml saline through the trachea, and samples were spun at 10,000 × *g*. To determine the amount of IFN I/III, IFN-sensitive epithelial cells from Mx2-Luc reporter mice were treated with the supernatant as described previously (46). A standard curve for the calculation of IFN concentrations was obtained by treating cells with serial dilutions of IFN-β.

### Flow cytometric analysis.

Cells were collected from BAL fluid, were incubated with a CD16/CD36 (2.4G2) antibody, and stained for CD11b (M1/70), Gr-1 (RB6-8C5), and F4/80 (BM8). Data were acquired using a BD LSRFortessa cell analyzer and analyzed using FlowJo (Tree Star).

### Microarray data accession number.

Microarray data were deposited in NCBI’s Gene Expression Omnibus and are accessible through the GEO series accession number GSE57008.

## SUPPLEMENTAL MATERIAL

Figure S1  Mortality, body weight loss, and viral load following respiratory IAV infection. (A and B) C57BL/6 mice were intranasally infected with influenza virus PR8/A/34(H1N1) and observed for body weight loss (A) and mortality (B). Weight loss and survival curves show results of *n* = 7 mice from two independent infection experiments. (C) The viral load in lung tissue was determined as nucleoprotein (NP) RNA copies by absolute quantitative real-time PCR (qRT-PCR). Perfused lung tissue was stored in RNAlater (Ambion), and RNA was extracted using the RNeasy kit (Qiagen). One microgram of RNA was used for cDNA synthesis using the Maxima First Strand cDNA synthesis kit for qRT-PCR (Thermo Scientific). Absolute qRT-PCR was performed on a LightCycler 480 II (Roche) using FastStart Essential DNA Green Master (Roche). Per reaction mixture, 125 ng reverse-transcribed RNA was used and compared to a plasmid standard containing defined copy numbers of the IAV nucleoprotein gene. NP primers were GAGGGGTGAGAATGGACGAAAAAC (5′-NP) and CAGGCAGGCAGGCAGGACTT (3′-NP) and were used in a final concentration of 500 nmol/liter. Data are shown for individual mice from two independent infection experiments. (D) Likewise, the number of NP RNA copies was determined for 37.5 ng of cDNA prepared from the AECII RNA samples isolated for the microarray analyses. (E) Flow cytometric analysis of sorted AECII. Dot plots are representative for ungated cells after sorting. (F) Total number of AECII isolated per mouse for the microarray experiments. Download Figure S1, PDF file, 0.4 MB

Figure S2  Quantitative real-time PCR results confirm transcriptional activation of AECII *in vivo*. WT mice were infected with IAV or treated with PBS and sacrificed 3 days later. Total RNA from sorted AECII (5 mice per independent sample) was isolated for quantitative real-time PCR analysis using primer pairs specific for *ACTB*, *CXCL5*, *CXCL10*, *IFIT2*, *IL6*, *IRF7*, *MX2*, *RSAD2*, and *USP18* (A). One microgram of total RNA was used for cDNA synthesis using the Maxima First Strand cDNA synthesis kit for qRT-PCR (Thermo Scientific). Reactive qRT-PCR was performed on a LightCycler 480 II (Roche) using FastStart Essential DNA Green Master (Roche). Per reaction mixture, 35.7 ng reverse-transcribed RNA was used. Gene expression was normalized to the housekeeping gene *ACTB*, and fold changes were calculated using the ΔΔ*C_p_* method with efficiency correction (B). Groups were compared by unpaired, two-sided *t* test; * indicates *P* < 0.05, ** indicates *P* < 0.01, and *** indicates *P* < 0.001. Download Figure S2, PDF file, 0.3 MB

Figure S3  Analysis of transcriptional regulation in AECII and lung tissue isolated from IAV-infected TLR7ko mice. TLR7-deficient mice were intranasally infected with IAV or treated with PBS and sacrificed 3 days postinfection. Total RNA was isolated from whole lungs (*n* = 3 individual replicates) and sorted AECII (*n* = 2 individual sample pools; 5 mice per sample pool) and subjected to microarray analysis. Data were analyzed by comparing day 3 IAV-infected versus uninfected control samples. (A) Scatter plots of regulated transcripts with a fold change of ≥±2 (threshold represented by the diagonal lines). Data represent normalized log_2_ signal intensities (averaged over replicates). The number of up- and downregulated transcripts is indicated. (B) Venn diagram comparing the regulated transcripts identified in panel A with respect to regulation in lung and/or AECII. (C) Scatter plot showing absolute log_2_ fold changes of the transcripts identified in panel A. Red dashed bisecting lines indicate equal fold changes. Gray lines indicate the fold change threshold of ±2. (D) Transcriptional data of the WT and TLR7ko AECII control samples were compared and revealed similar baseline gene expression levels in the two mouse strains. The scatter plot shows the absolute log_2_ signal intensities. The defined fold change threshold of ±2 for transcriptional up- or downregulation is indicated by the diagonal lines. Download Figure S3, PDF file, 0.4 MB

Figure S4  The differential expression of molecules involved in antimicrobial defense is blunted in IAV-infected TLR7ko mice. The graphs depict the fold change regulation of selected transcripts as determined by microarray analysis of AECII and lungs isolated from wild-type (WT) and TLR7ko mice at the indicated time points post-IAV infection. The graphs show the mean and individual results from two replicate microarray experiments (2 independent samples; 5 mice per sample) for AECII and three replicate microarray experiments for lung tissue (three independent samples). The transcripts listed are grouped into those encoding pathogen recognition receptors (A), factors associated with the IFN I/III response (B), cytokines and chemokines (C), and factors associated with antigen presentation (D). For each bar graph, the dashed horizontal line indicates a fold change of 2. Download Figure S4, PDF file, 0.2 MB

Figure S5  Macrophages, PMN, and IFN I/III in bronchoalveolar lavage fluid of TLR7ko mice. Wild-type (WT) mice and TLR7ko mice were sacrificed at the indicated time points post-IAV infection. Bronchoalveolar lavage (BAL) fluid cells were counted (A), and the macrophage and polymorphonuclear cell (PMN) populations (B) were assessed by flow cytometry and are shown as means ± standard errors of the means (SEM). Cell populations were analyzed by gating on macrophages (F4/80^+^ cells) within all acquired cells and gating on PMN (Gr-1^+^/CD11b^+^) within the F4/80^−^ cell fraction. Macrophage and PMN numbers (C) were calculated from the absolute cell count and percent population for all analyzed individual mice and are shown as individual mice and mean per group. (D) Bioactive IFN I/III in BAL fluid was assessed and is shown as mean ± SEM. All data are compiled from at least two independent infection experiments with *n* ≥ 5 mice/group and were compared by unpaired, two-sided *t* test (* indicates *P* value of <0.05; ** indicates *P* value of <0.005; *** indicates *P* value of <0.001). Download Figure S5, PDF file, 0.2 MB

Figure S6  Mortality, body weight loss, and viral load following respiratory IAV infection of TLR7ko mice. (A and B) Wild-type (WT) and TLR7ko mice were intranasally infected with influenza virus PR8/A/34(H1N1) and observed for body weight loss (A) and mortality (B). Weight loss and survival curves show results of *n* = 7 WT and *n* = 10 TLR7ko mice from two independent infection experiments. (C) The viral load in lung tissue was determined as nucleoprotein (NP) RNA copies by absolute qRT-PCR. Perfused lung tissue was stored in RNAlater (Ambion), and RNA was extracted using the RNeasy kit (Qiagen). One microgram of RNA was used for cDNA synthesis using the Maxima First Strand cDNA synthesis kit for qRT-PCR (Thermo Scientific). Absolute qRT-PCR was performed on a LightCycler 480 II (Roche) using FastStart Essential DNA Green Master (Roche). Per reaction mixture, 125 ng reverse-transcribed RNA was used and compared to a plasmid standard containing defined copy numbers of the IAV nucleoprotein gene. NP primers were GAGGGGTGAGAATGGACGAAAAAC (5′-NP) and CAGGCAGGCAGGCAGGACTT (3′-NP) and were used in a final concentration of 500 nmol/liter. Data are shown for individual mice from two independent infection experiments. (D) Likewise, the number of NP RNA copies was determined for 37.5 ng of cDNA prepared from the AECII RNA samples isolated for the microarray analyses. (E) Number of AECII per mouse isolated for the microarray and PCR experiments. Download Figure S6, PDF file, 0.2 MB

Table S1  The 50 most intensely upregulated transcripts dependent on TLR7 expression. The table lists the 50 most intensely upregulated TLR7-dependent transcripts differentially expressed only in AECII of wild-type but not TLR7ko mice following *in vivo* influenza A virus infection. Transcripts are ranked by fold change regulation on day 3 postinfection. Table S1, PDF file, 0.02 MB

Table S2  Top 10 functional pathways most significantly overrepresented in the transcripts differentially expressed in TLR7ko AECII following IAV. Overrepresentation of functional pathways in the transcripts differentially regulated in AECII isolated from IAV-infected WT and TLR7ko mice during the first 3 days following infection was assessed using the Ingenuity pathway analysis tool. The 10 pathways most significantly overrepresented in the transcripts differentially regulated in TLR7ko AECII on day 3 postinfection are listed. Pathways were ranked by Fisher exact test *P* value. The table indicates the −log(*P* value) for overrepresentation of the respective pathways for all microarray data sets (WT and TLR7ko AECII; days 1, 2, and 3 postinfection), listing only those values indicating statistical significance (*P* < 0.05). Table S2, PDF file, 0.02 MB
