# Supplementary material for: Alveolar Type II Epithelial Cells Contribute to the Anti-Influenza A Virus Response in the Lung by Integrating Pathogen- and Microenvironment-Derived Signals
Source: mBio. 2016 May 3;7(3):e00276-16. doi: 10.1128/mBio.00276-16 (PMC4959657; doi:10.1128/mBio.00276-16)
Supplement: Figure S1 — Mortality, body weight loss, and viral load following respiratory IAV infection. (A and B) C57BL/6 mice were intranasally infected with influenza virus PR8/A/34(H1N1) and observed for body weight loss (A) and mortality (B). Weight loss and survival curves show results of n = 7 mice from two independent infection experiments. (C) The viral load in lung tissue was determined as nucleoprotein (NP) RNA copies by absolute quantitative real-time PCR (qRT-PCR). Perfused lung tissue was stored in RNAlater (Ambion), and RNA was extracted using the RNeasy kit (Qiagen). One microgram of RNA was used for cDNA synthesis using the Maxima First Strand cDNA synthesis kit for qRT-PCR (Thermo Scientific). Absolute qRT-PCR was performed on a LightCycler 480 II (Roche) using FastStart Essential DNA Green Master (Roche). Per reaction mixture, 125 ng reverse-transcribed RNA was used and compared to a plasmid standard containing defined copy numbers of the IAV nucleoprotein gene. NP primers were GAGGGGTGAGAATGGACGAAAAAC (5′-NP) and CAGGCAGGCAGGCAGGACTT (3′-NP) and were used in a final concentration of 500 nmol/liter. Data are shown for individual mice from two independent infection experiments. (D) Likewise, the number of NP RNA copies was determined for 37.5 ng of cDNA prepared from the AECII RNA samples isolated for the microarray analyses. (E) Flow cytometric analysis of sorted AECII. Dot plots are representative for ungated cells after sorting. (F) Total number of AECII isolated per mouse for the microarray experiments. Download [file mbo002162795sf1.pdf]

Figure S1

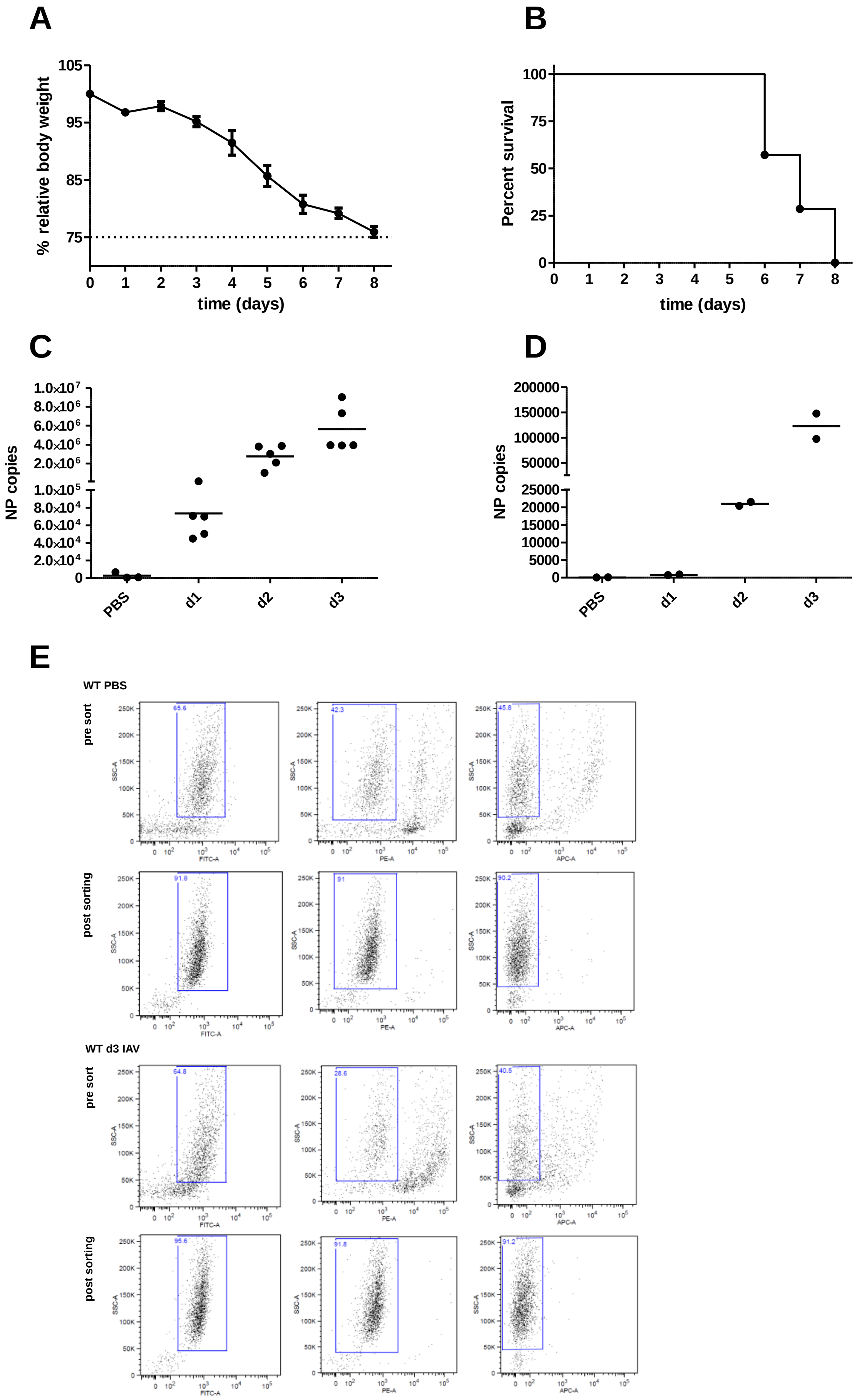

A
% relative body weight
105
95
85
75
0 1 2 3 4 5 6 7 8
time (days)
B
Percent survival
100
75
50
25
0
0 1 2 3 4 5 6 7 8
time (days)
C
NP copies
PBS
d1
d2
d3
D
NP copies
200000
150000
100000
50000
25000
20000
15000
10000
5000
0
PBS
d1
d2
d3
E
WT PBS
pre sort
post sorting
WT d3 IAV
pre sort
post sorting
SSC-A
FITC-A
PE-A
APC-A

F

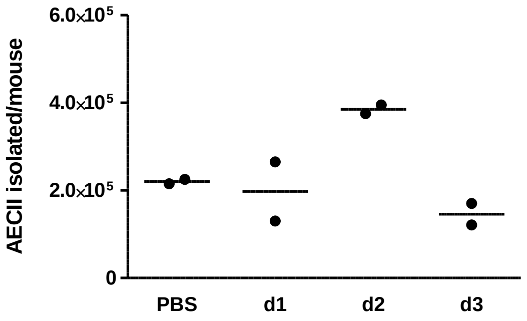

Figure S1: Mortality, body weight loss and viral load following respiratory IAV-infection. C57BL/6 mice were intranasally infected with influenza virus PR8/A/34(H1N1) and observed for body-weight loss (A) and mortality (B). Weight loss and survival curves show results of n = 7 mice from two independent infection experiments. C) The viral load in lung tissue was determined as nucleoprotein (NP) RNA-copies by absolute qRT-PCR. Perfused lung tissue was stored in RNAlater (Ambion) and RNA was extracted using the RNA-easy kit (Qiagen). 1 µg RNA was used for cDNA synthesis using the Maxima First Strand cDNA Synthesis Kit for RT-qPCR (Thermo Scientific). Absolute qRT-PCR was perfomed on a LightCycler 480 II (Roche) using FastStart Essential DNA Green Master (Roche). Per reaction 125 ng reversely transcribed RNA was used and compared to a plasmid standard containing defined copy numbers of the IAV nucleoprotein gene. NP-primers were GAGGGGTGAGAATGGACGAAAAAC (5'-NP) and CAGGCAGGCAGGCAGGACTT (3'-NP) and were used in a final concentration of 500 nmol/l. Data are shown for individual mice from two independent infection experiments. D) Likewise, the number of NP RNA-copies was determined for 37.5 ng of cDNA prepared from the AECII RNA samples isolated for the microarray analyses. E) Flow cytometric analysis of sorted AECII. Dot plots are representative for ungated cells after sorting. F) The total number of AECII isolated per mouse for the microarray experiments.
