# Supplementary material for: Alveolar Type II Epithelial Cells Contribute to the Anti-Influenza A Virus Response in the Lung by Integrating Pathogen- and Microenvironment-Derived Signals
Source: mBio. 2016 May 3;7(3):e00276-16. doi: 10.1128/mBio.00276-16 (PMC4959657; doi:10.1128/mBio.00276-16)
Supplement: Figure S2 — Quantitative real-time PCR results confirm transcriptional activation of AECII in vivo. WT mice were infected with IAV or treated with PBS and sacrificed 3 days later. Total RNA from sorted AECII (5 mice per independent sample) was isolated for quantitative real-time PCR analysis using primer pairs specific for ACTB, CXCL5, CXCL10, IFIT2, IL6, IRF7, MX2, RSAD2, and USP18 (A). One microgram of total RNA was used for cDNA synthesis using the Maxima First Strand cDNA synthesis kit for qRT-PCR (Thermo Scientific). Reactive qRT-PCR was performed on a LightCycler 480 II (Roche) using FastStart Essential DNA Green Master (Roche). Per reaction mixture, 35.7 ng reverse-transcribed RNA was used. Gene expression was normalized to the housekeeping gene ACTB, and fold changes were calculated using the ΔΔCp method with efficiency correction (B). Groups were compared by unpaired, two-sided t test; * indicates P < 0.05, ** indicates P < 0.01, and *** indicates P < 0.001. Download [file mbo002162795sf2.pdf]

Figure S2

A

Gene		forward primer	reverse primer	
actb	5'	CTTCTTTGCAGCTCCTTCGT	TCCTTCTGACCCATTCCCAC	3'
cxcl5	5'	CCGCTGGCATTTCTGTTG	ATGACTTCCACCGTAGGGCACTGT	3'
cxcl10	5'	GCTGCCGTCATTTTCTGC	TCTCACTGGCCCGTCATC	3'
ifit2	5'	CACCTTCGGTATGGCAACTT	GCAAGGCCTCAGAATCAGAC	3'
il6	5'	ACCACGGCCTTCCCTACTTC	GCCATTGCACAACTCTTTTCTC	3'
irf7	5'	GAAGACCCTGATCCTGGTGA	CCAGGTCCATGAGGAAGTGT	3'
mx2	5'	TCACCAGAGTGCAAGTGAGG	CATTCTCCCTCTGCCACATT	3'
rsad2	5'	GTCCTGTTTGGTGCCTGAAT	GCCACGCTTCAGAAACATCT	3'
usp-18	5'	AAGGACCAGATCACGGACAC	CATCCTCCAGGGTTTTCAGA	3'

B

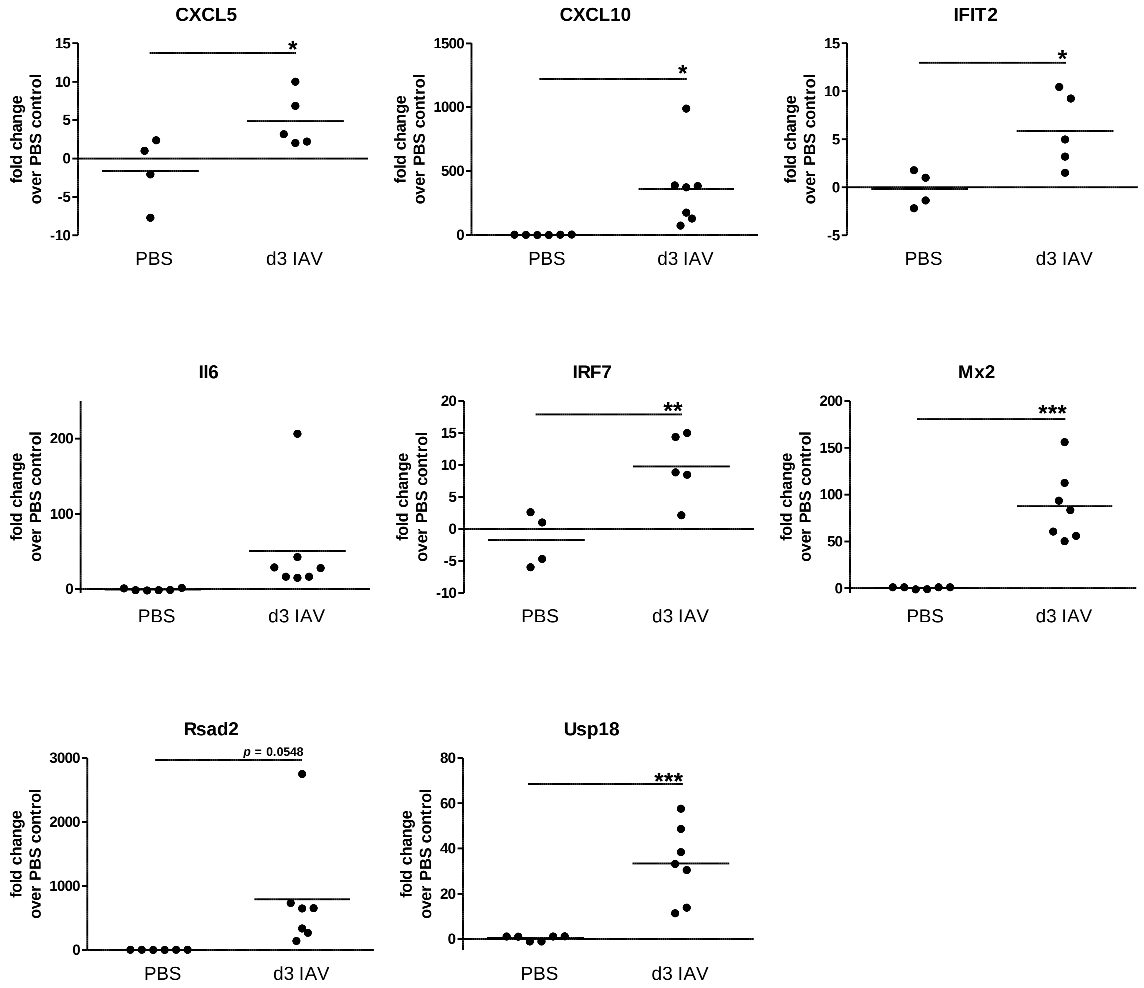

Figure S2: Quantitative real-time PCR results confirm transcriptional activation of AECII *in vivo*. WT mice were infected with IAV or treated with PBS and sacrificed three days later. Total RNA from sorted AECII (5 mice per independent sample) was isolated for quantitative real-time PCR analysis using primer pairs specific for *actb*, *cxcl5*, *cxcl10*, *ifit2*, *il6*, *irf7*, *mx2*, *rsad2*, *usp18* (A). 1 µg total RNA was used for cDNA synthesis using the Maxima First Strand cDNA Synthesis Kit for RT-qPCR (Thermo Scientific). Reative qRT-PCR was perfomed on a LightCycler 480 II (Roche) using FastStart Essential DNA Green Master (Roche). Per reaction 35.7 ng reversely transcribed RNA was used. Gene expression was normalized to the housekeeping gene *actb* and fold changes were calculated using the $\Delta\Delta C_p$ method with efficiency correction (B). Groups were compared by unpaired, two-sided t-test and * indicates $p < 0.05$, ** indicates $p < 0.01$ and *** indicates $p < 0.001$.
