# Supplementary material for: Alveolar Type II Epithelial Cells Contribute to the Anti-Influenza A Virus Response in the Lung by Integrating Pathogen- and Microenvironment-Derived Signals
Source: mBio. 2016 May 3;7(3):e00276-16. doi: 10.1128/mBio.00276-16 (PMC4959657; doi:10.1128/mBio.00276-16)
Supplement: Figure S3 — Analysis of transcriptional regulation in AECII and lung tissue isolated from IAV-infected TLR7ko mice. TLR7-deficient mice were intranasally infected with IAV or treated with PBS and sacrificed 3 days postinfection. Total RNA was isolated from whole lungs (n = 3 individual replicates) and sorted AECII (n = 2 individual sample pools; 5 mice per sample pool) and subjected to microarray analysis. Data were analyzed by comparing day 3 IAV-infected versus uninfected control samples. (A) Scatter plots of regulated transcripts with a fold change of ≥±2 (threshold represented by the diagonal lines). Data represent normalized log2 signal intensities (averaged over replicates). The number of up- and downregulated transcripts is indicated. (B) Venn diagram comparing the regulated transcripts identified in panel A with respect to regulation in lung and/or AECII. (C) Scatter plot showing absolute log2 fold changes of the transcripts identified in panel A. Red dashed bisecting lines indicate equal fold changes. Gray lines indicate the fold change threshold of ±2. (D) Transcriptional data of the WT and TLR7ko AECII control samples were compared and revealed similar baseline gene expression levels in the two mouse strains. The scatter plot shows the absolute log2 signal intensities. The defined fold change threshold of ±2 for transcriptional up- or downregulation is indicated by the diagonal lines. Download [file mbo002162795sf3.pdf]

Figure S3

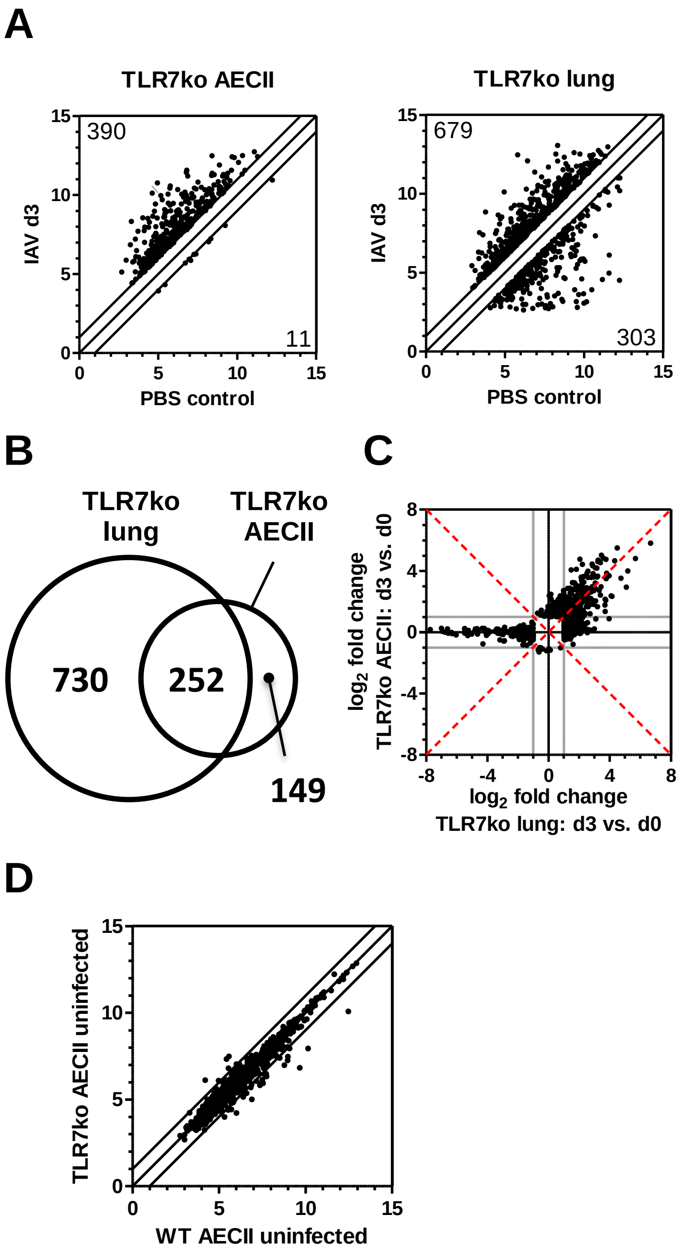

Figure S3: Analysis of transcriptional regulation in AECII and lung tissue isolated from IAV-infected TLR7ko mice.

TLR7-deficient mice were intranasally infected with IAV or treated with PBS and sacrificed three days post infection. Total RNA was isolated from whole lungs (n = 3 independent replicates) and sorted AECII (n = 2 independent sample pools; 5 mice per sample pool) and subjected to microarray analysis. Data were analyzed by comparing d3 IAV-infected vs. uninfected control samples. A) Scatter plots of regulated transcripts with a fold change ≥ ±2 (threshold represented by the diagonal lines). Data represent normalized log_2 signal intensities (averaged over replicates).

The number of up- und down-regulated transcripts is indicated. B) Venn diagram comparing the regulated transcripts identified in A with respect to regulation in lung and/or AECII. C) Scatter plot showing absolute log_2 fold-changes of the transcripts identified in A. Red dashed bisecting lines indicate equal fold changes. Grey lines indicate the fold-change threshold of ±2. D) Transcriptional data of the WT and TLR7ko AECII control samples was compared and revealed similar baseline gene expression in both mouse strains. The scatter plot shows the absolute log_2 signal intensities. The defined fold-change threshold of ±2 for transcriptional up- or down-regulation is indicated by the diagonal lines.
