# Supplementary material for: Alveolar Type II Epithelial Cells Contribute to the Anti-Influenza A Virus Response in the Lung by Integrating Pathogen- and Microenvironment-Derived Signals
Source: mBio. 2016 May 3;7(3):e00276-16. doi: 10.1128/mBio.00276-16 (PMC4959657; doi:10.1128/mBio.00276-16)
Supplement: Figure S4 — The differential expression of molecules involved in antimicrobial defense is blunted in IAV-infected TLR7ko mice. The graphs depict the fold change regulation of selected transcripts as determined by microarray analysis of AECII and lungs isolated from wild-type (WT) and TLR7ko mice at the indicated time points post-IAV infection. The graphs show the mean and individual results from two replicate microarray experiments (2 independent samples; 5 mice per sample) for AECII and three replicate microarray experiments for lung tissue (three independent samples). The transcripts listed are grouped into those encoding pathogen recognition receptors (A), factors associated with the IFN I/III response (B), cytokines and chemokines (C), and factors associated with antigen presentation (D). For each bar graph, the dashed horizontal line indicates a fold change of 2. Download [file mbo002162795sf4.pdf]

Figure S4

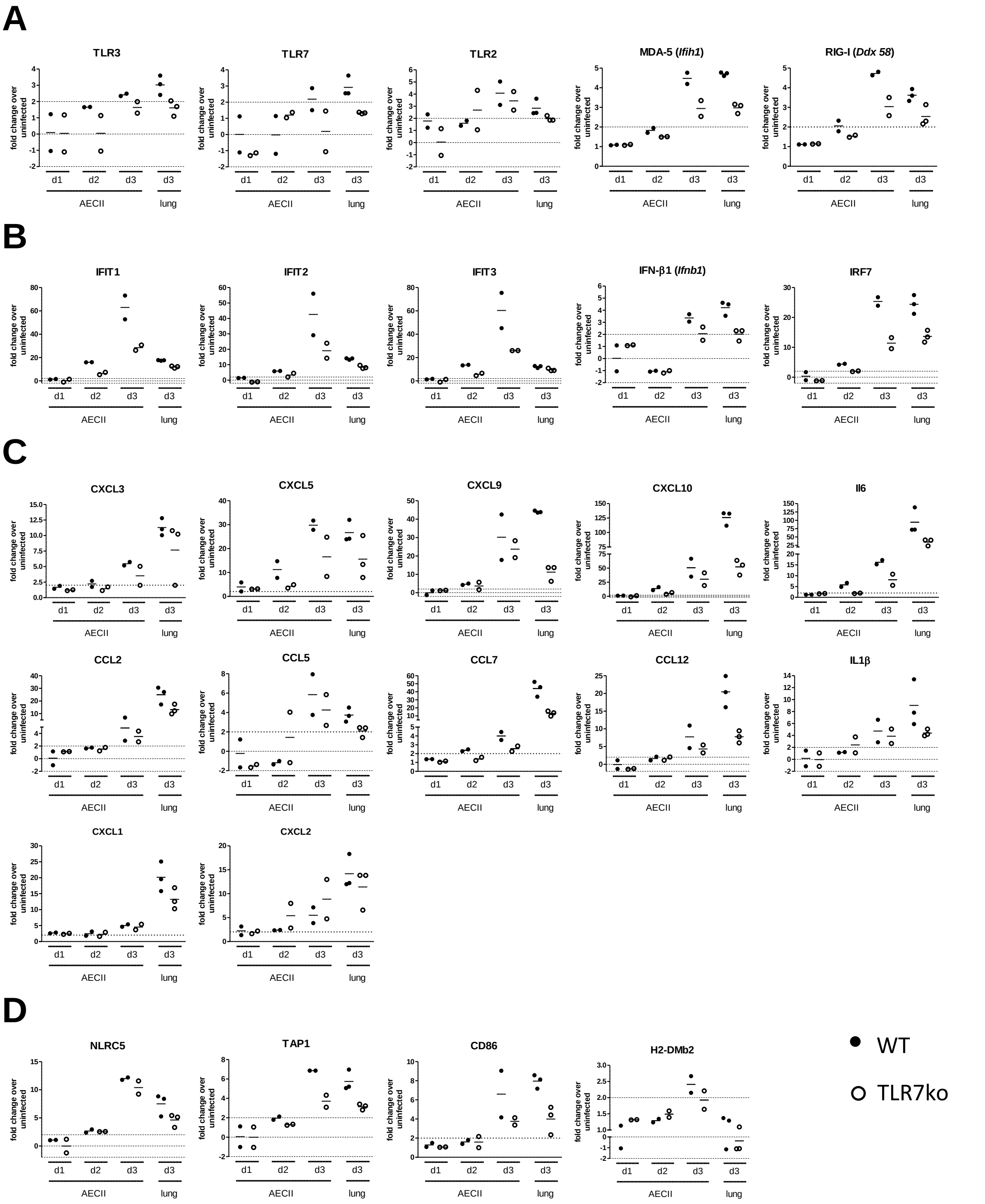

Figure S4: The differential expression of molecules involved in anti-microbial defense is blunted in IAV-infected TLR7ko mice.

The graphs depict the fold-change regulation of selected transcripts as determined by microarray analysis of AECII and lungs isolated from wild-type (WT) and TLR7ko mice at the indicated time-points post IAV-infection. The graphs show the mean and individual results of two replicate microarray experiments (2 independent samples; 5 mice per sample) for AECII and three replicate microarray experiments for lung tissue (three independent samples). The transcripts listed are grouped into those encoding pathogen recognition receptors (A), factors associated with the IFN I/III response (B), cytokines and chemokines (C) and factors associated with antigen-presentation (D). For each bar graph, the dashed horizontal line indicates a fold change of 2.
