# Supplementary material for: Alveolar Type II Epithelial Cells Contribute to the Anti-Influenza A Virus Response in the Lung by Integrating Pathogen- and Microenvironment-Derived Signals
Source: mBio. 2016 May 3;7(3):e00276-16. doi: 10.1128/mBio.00276-16 (PMC4959657; doi:10.1128/mBio.00276-16)
Supplement: Figure S5 — Macrophages, PMN, and IFN I/III in bronchoalveolar lavage fluid of TLR7ko mice. Wild-type (WT) mice and TLR7ko mice were sacrificed at the indicated time points post-IAV infection. Bronchoalveolar lavage (BAL) fluid cells were counted (A), and the macrophage and polymorphonuclear cell (PMN) populations (B) were assessed by flow cytometry and are shown as means ± standard errors of the means (SEM). Cell populations were analyzed by gating on macrophages (F4/80+ cells) within all acquired cells and gating on PMN (Gr-1+/CD11b+) within the F4/80− cell fraction. Macrophage and PMN numbers (C) were calculated from the absolute cell count and percent population for all analyzed individual mice and are shown as individual mice and mean per group. (D) Bioactive IFN I/III in BAL fluid was assessed and is shown as mean ± SEM. All data are compiled from at least two independent infection experiments with n ≥ 5 mice/group and were compared by unpaired, two-sided t test (* indicates P value of <0.05; ** indicates P value of <0.005; *** indicates P value of <0.001). Download [file mbo002162795sf5.pdf]

Figure S5

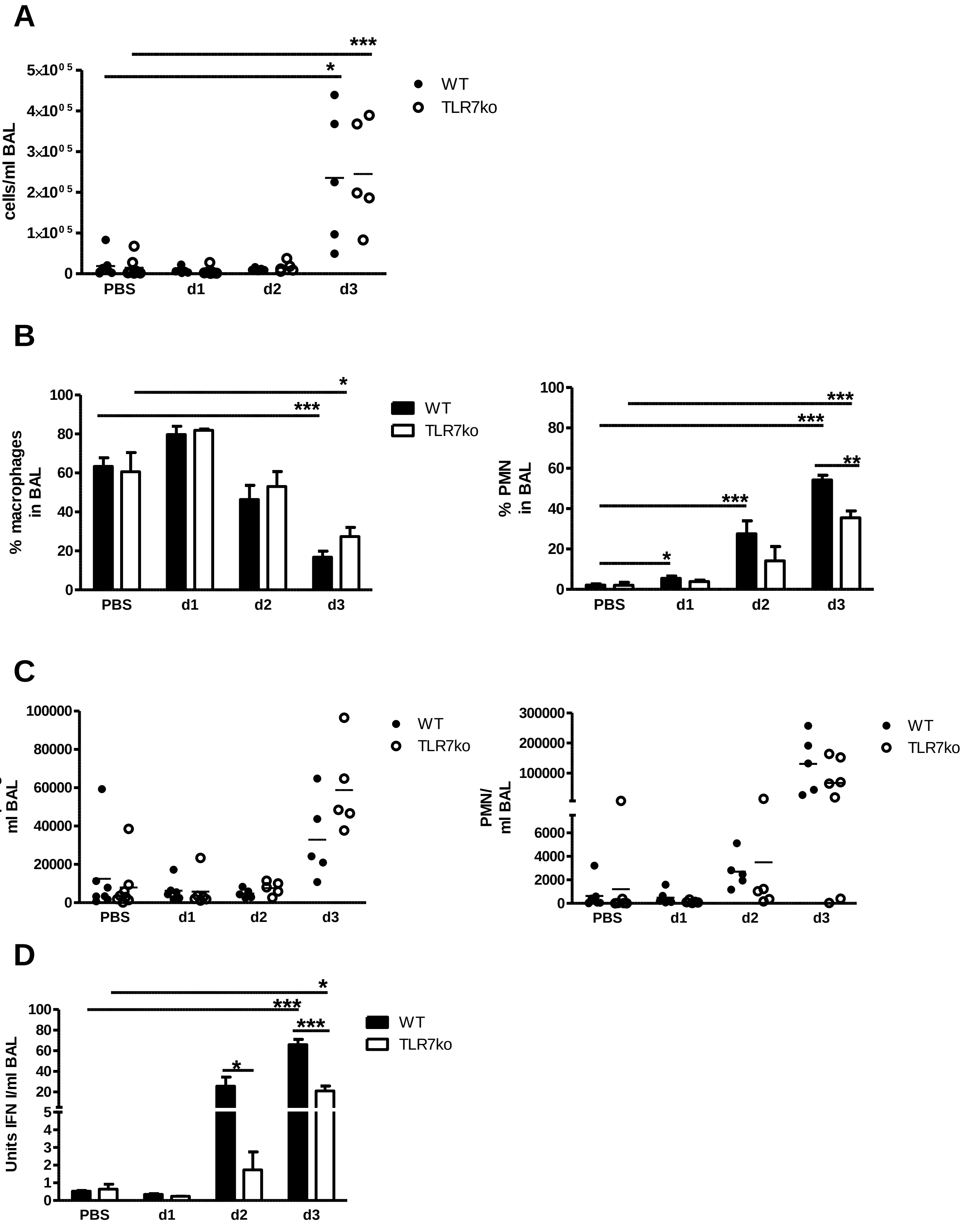
A
cells/ml BAL
5x10^5
4x10^5
3x10^5
2x10^5
1x10^5
0
PBS
d1
d2
d3
***
*
WT
TLR7ko
B
% macrophages in BAL
100
80
60
40
20
0
PBS
d1
d2
d3
*
***
WT
TLR7ko
% PMN in BAL
100
80
60
40
20
0
PBS
d1
d2
d3
***
***
**
***
*
C
macrophages/ ml BAL
100000
80000
60000
40000
20000
0
PBS
d1
d2
d3
WT
TLR7ko
PMN/ ml BAL
300000
200000
100000
6000
4000
2000
0
PBS
d1
d2
d3
WT
TLR7ko
D
Units IFN I/ml BAL
100
80
60
40
20
5
4
3
2
1
0
PBS
d1
d2
d3
*
***
***
*
WT
TLR7ko

Figure S5: Macrophages, PMN and IFNI/III in bronchoalveolar lavage of TLR7ko mice.

Wild-type (WT) mice and TLR7ko mice were sacrificed at the indicated time points post IAV-infection. Bronchoalveolar lavage (BAL) cells were counted (A) and the macrophage and polymorphonuclear cell (PMN) populations (B) were assessed by flow cytometry and are shown as mean ± SEM. Cell populations were analyzed by gating on macrophages (F4/80+ cells) within all acquired cells and gating on PMN (Gr1+/CD11b+) within the F4/80¬- cell fraction. Macrophage and PMN numbers (C) were calculated from the absolute cell count and percent population for all analyzed individual mice and are shown as individual mice and mean/group. (D) Bioactive IFNI/III in BAL was assessed and is shown as mean ± SEM. All data are compiled from at least two independent infection experiments with n ≥ 5 mice/group and were compared by unpaired, two-sided t-test (* indicates p-values < 0.05; ** indicates p-values < 0.005; *** indicates p-values < 0.001).
