# Supplementary material for: Alveolar Type II Epithelial Cells Contribute to the Anti-Influenza A Virus Response in the Lung by Integrating Pathogen- and Microenvironment-Derived Signals
Source: mBio. 2016 May 3;7(3):e00276-16. doi: 10.1128/mBio.00276-16 (PMC4959657; doi:10.1128/mBio.00276-16)
Supplement: Figure S6 — Mortality, body weight loss, and viral load following respiratory IAV infection of TLR7ko mice. (A and B) Wild-type (WT) and TLR7ko mice were intranasally infected with influenza virus PR8/A/34(H1N1) and observed for body weight loss (A) and mortality (B). Weight loss and survival curves show results of n = 7 WT and n = 10 TLR7ko mice from two independent infection experiments. (C) The viral load in lung tissue was determined as nucleoprotein (NP) RNA copies by absolute qRT-PCR. Perfused lung tissue was stored in RNAlater (Ambion), and RNA was extracted using the RNeasy kit (Qiagen). One microgram of RNA was used for cDNA synthesis using the Maxima First Strand cDNA synthesis kit for qRT-PCR (Thermo Scientific). Absolute qRT-PCR was performed on a LightCycler 480 II (Roche) using FastStart Essential DNA Green Master (Roche). Per reaction mixture, 125 ng reverse-transcribed RNA was used and compared to a plasmid standard containing defined copy numbers of the IAV nucleoprotein gene. NP primers were GAGGGGTGAGAATGGACGAAAAAC (5′-NP) and CAGGCAGGCAGGCAGGACTT (3′-NP) and were used in a final concentration of 500 nmol/liter. Data are shown for individual mice from two independent infection experiments. (D) Likewise, the number of NP RNA copies was determined for 37.5 ng of cDNA prepared from the AECII RNA samples isolated for the microarray analyses. (E) Number of AECII per mouse isolated for the microarray and PCR experiments. Download [file mbo002162795sf6.pdf]

Figure S6

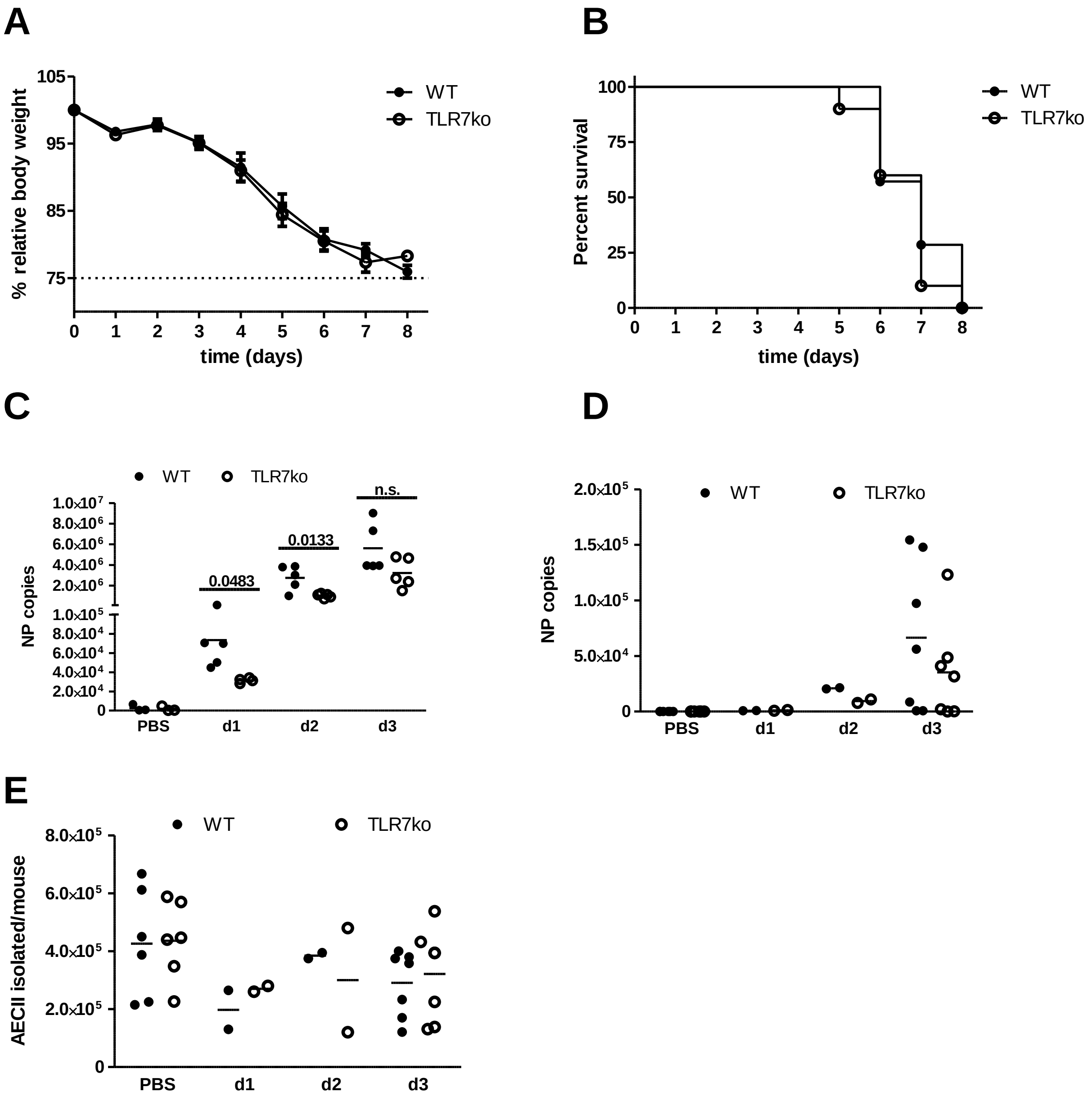

Figure S6: Mortality, body weight loss and viral load following respiratory IAV-infection of TLR7ko mice. Wild- type (WT) and TLR7ko mice were intranasally infected with influenza virus PR8/A/34(H1N1) and observed for body-weight loss (A) and mortality (B). Weight loss and survival curves show results of n = 7 WT and n = 10 TLR7ko mice from two independent infection experiments. (C) The viral load in lung tissue was determined as nucleoprotein (NP) RNA-copies by absolute qRT-PCR. Perfused lung tissue was stored in RNAlater (Ambion) and RNA was extracted using the RNA-easy kit (Qiagen). 1 µg RNA was used for cDNA synthesis using the

Maxima First Strand cDNA Synthesis Kit for RT-qPCR (Thermo Scientific). Absolute qRT-PCR was perfomed on a LightCycler 480 II (Roche) using FastStart Essential DNA Green Master (Roche). Per reaction 125 ng reversely transcribed RNA was used and compared to a plasmid standard containing defined copy numbers of the IAV nucleoprotein gene. NP-primers were GAGGGGTGAGAATGGACGAAAAAC (5'-NP) and CAGGCAGGCAGGCAGGACTT (3'-NP) and were used in a final concentration of 500 nmol/l. Data are shown for individual mice from two independent infection experiments. (D) Likewise, the number of NP RNA-copies was determined for 37.5 ng of cDNA prepared from the AECII RNA samples isolated for the microarray analyses. (E) The number of AECII per mouse isolated for the microarray and PCR experiments.
