# Supplementary material for: Alveolar Type II Epithelial Cells Contribute to the Anti-Influenza A Virus Response in the Lung by Integrating Pathogen- and Microenvironment-Derived Signals
Source: mBio. 2016 May 3;7(3):e00276-16. doi: 10.1128/mBio.00276-16 (PMC4959657; doi:10.1128/mBio.00276-16)
Supplement: Table S1 — The 50 most intensely upregulated transcripts dependent on TLR7 expression. The table lists the 50 most intensely upregulated TLR7-dependent transcripts differentially expressed only in AECII of wild-type but not TLR7ko mice following in vivo influenza A virus infection. Transcripts are ranked by fold change regulation on day 3 postinfection. [file mbo002162795st1.pdf]

Table S1

Name	FC WT AECII d3
BC037703	***4.0***
Tor3a	***3.6***
Rgs16	***3.6***
C1ra	***3.5***
Xaf1	***3.5***
Ms4a4d	***3.4***
Spats2l	***3.4***
Ifnb1	***3.3***
Timp1	***3.3***
Mitd1	***3.3***
Tlr3	***3.3***
Gm14085 /// Slc28a2	***3.2***
Cxcl13	***3.1***
Nmi	***3.1***
Tor3a	***3.1***
Nampt	***3.0***
Cd40	***3.0***
Runx3	***3.0***
LOC100503912	***3.0***
Ifi35	***3.0***
H2-D1	***3.0***
Steap4	***2.9***
Adora3	***2.9***
Aim1	***2.9***
Vnn1	***2.9***
Trim21	***2.8***
E030037K03Rik	***2.8***
Tlr1	***2.8***
Pfkfb3	***2.8***
AW011738	***2.7***
Ly6c1 /// Ly6c2	***2.7***
Cyp4f18	***2.7***
Isg20	***2.7***
Cd83	***2.7***
Hap1	***2.7***
Daxx	***2.7***
Ddx60	***2.7***
Slc1a1	***2.7***
Vcam1	***2.7***
Tor3a	***2.7***
Ptpro	***2.6***
Adar	***2.6***
Spats2l	***2.6***
Cd14	***2.6***
Cp	***2.6***
Gatm	***2.6***
Pf4	***2.6***
Trim21	***2.6***
Cxcl14	***2.6***
Agrn	***2.5***

Table S1: The fifty most intensely up-regulated transcripts dependent on TLR7-expression. The table lists the fifty most intensely up-regulated TLR7-dependent transcripts differentially expressed only in AECII of wild-type but not TLR7ko mice following *in vivo* influenza A virus infection. Transcripts are ranked by fold-change regulation on day three post infection.
