# Supplementary material for: Alveolar Type II Epithelial Cells Contribute to the Anti-Influenza A Virus Response in the Lung by Integrating Pathogen- and Microenvironment-Derived Signals
Source: mBio. 2016 May 3;7(3):e00276-16. doi: 10.1128/mBio.00276-16 (PMC4959657; doi:10.1128/mBio.00276-16)
Supplement: Table S2 — Top 10 functional pathways most significantly overrepresented in the transcripts differentially expressed in TLR7ko AECII following IAV. Overrepresentation of functional pathways in the transcripts differentially regulated in AECII isolated from IAV-infected WT and TLR7ko mice during the first 3 days following infection was assessed using the Ingenuity pathway analysis tool. The 10 pathways most significantly overrepresented in the transcripts differentially regulated in TLR7ko AECII on day 3 postinfection are listed. Pathways were ranked by Fisher exact test P value. The table indicates the −log(P value) for overrepresentation of the respective pathways for all microarray data sets (WT and TLR7ko AECII; days 1, 2, and 3 postinfection), listing only those values indicating statistical significance (P < 0.05). [file mbo002162795st2.pdf]

Table S2

Ranking by *p*-value		Canonical Pathway	AECII d1		AECII d2		AECII d3	
TLR7ko	WT		WT	TLR7ko	WT	TLR7ko	WT	TLR7ko
1	1	Communication between Innate and Adaptive Immune Cells	1.53		2.05	5.41	20.49	15.40
2	3	Granulocyte Adhesion and Diapedesis	2.53		3.31	8.63	15.44	14.83
3	2	Role of Pattern Recognition Receptors in Recognition of Bacteria and Viruses			3.03	4.47	19.04	13.85
4	4	Crosstalk between Dendritic Cells and Natural Killer Cells					14.79	12.42
5	11	Activation of IRF by Cytosolic Pattern Recognition Receptors			6.82	1.88	11.62	12.30
6	7	Agranulocyte Adhesion and Diapedesis	2.44		3.14	6.36	12.91	12.27
7	13	Role of Hypercytokinemia/hyperchemokinemia in the Pathogenesis of Influenza			1.35	5.18	10.22	11.73
8	9	Interferon Signaling			4.56	2.52	12.64	11.32
9	5	Dendritic Cell Maturation				3.14	14.33	10.80
10	10	Antigen Presentation Pathway			2.34		11.66	10.21

Table S2: Top-ten functional pathways most significantly over represented in the transcripts differentially expressed in TLR7ko AECII following IAV. Over representation of functional pathways in the transcripts differentially regulated in AECII isolated from IAV-infected WT and TLR7ko mice during the first three days following infection was assessed using the Ingenuity Pathway Analysis tool. The ten pathways most significantly over represented in the transcripts differentially regulated in TLR7ko AECII on day 3 post infection are listed. Pathways were ranked by Fisher Exact test *p*-value. The table indicates the -log(*p*-value) for over

representation of the respective pathways for all microarray data-sets (WT and TLR7ko AECII; days one, two and three post infection), listing only those values indicating statistical significance ($p < 0.05$).
